# “The Old Ways Are the Old Ways”: A Qualitative Study of Chinese Medicine Care in Indigenous Communities in Canada

**DOI:** 10.1089/jicm.2022.0646

**Published:** 2023-06-06

**Authors:** Avery McNair, Nadine Ijaz

**Affiliations:** ^1^ School of Population and Public Health, University of British Columbia, Vancouver, Canada.; ^2^ Department of Law and Legal Studies, Faculty of Public Affairs, Carleton University, Ottawa, Canada.

**Keywords:** indigenous. Medicine, traditional, Traditional Chinese Medicine, acupuncture, transcultural studies, cultural humility

## Abstract

**Objectives:**

Owing to colonization's impacts, Indigenous Peoples in Canada face a disproportionate share of health challenges and suffer inequitable access to health care today. In recent years, an increasing number of Indigenous-led health services have emerged, which—informed by decolonial principles, including “culture-as-cure”—holistically center local Indigenous cultural, spiritual, and healing knowledges and practices. Aligned with decolonial principles, this work examines the delivery of Chinese Medicine (CM) care—an East Asian Indigenous therapeutic approach—in Indigenous communities in British Columbia, Canada.

**Design:**

Informed by qualitative interviews with three licensed CM practitioners and one biomedical clinician working in such clinics, the work provides a descriptive account of clinical operations, and thematically explores operational successes and challenges.

**Results:**

Four CM clinical programs were identified, all operating on First Nations reserves, including settings at multidisciplinary community health centers, a First Nation Band Council office, and a school gymnasium. Most CM care was delivered free of charge, funded variously by nonprofit agency donations and provincial government reimbursement. Three central themes emerged across the study interviews. The first, transculturalism, emphasizes the conceptual overlap between CM and Indigenous belief systems in the Canadian context, which participants described as a source of strength in building trust for CM care as a nonlocal Indigenous therapeutic approach. The second theme, Cultural Humility, characterizes non-Indigenous practitioners' respectful outlook as guests on Indigenous land, taking community members' lead as to how they might best serve. The final theme, Multidimensional Healing, explores the physical, mental, and emotional healing that practitioners witnessed across their work.

**Conclusions:**

Despite economic and logistical challenges, study respondents expressed optimism about the potential for similar traditional medicine clinics to provide culturally resonant primary care in other underserved communities. Further research to learn about the experiences of First Nations community members receiving CM care is warranted.

## Introduction

Indigenous Peoples in Canada face more significant health challenges than their non-Indigenous counterparts, stemming from the far-reaching, intergenerational impacts of European colonization.^1–4^ These impacts include the ongoing enforcement of colonial policies (such as Canada's “Indian Act”) and, from the mid-1800s and in many cases continuing to the present day, extensive “violence, cultural genocide, legislated segregation, appropriation of lands, and social and economic oppression.”^1(p1)^ As a result, First Nations, Inuit and Métis communities in Canada suffer disproportionately high rates of infant mortality and premature death, disease, and disability^1,3,4^; face an “uneven distribution of health funding, resources, and services”^1(p26)^; and, experience widespread, racist discrimination within Canada's dominant health care system.^5,6^

Furthermore, biomedicine—which has played an instrumental role as a tool of empire across nations impacted by colonization—remains the dominant, state-sanctioned form of health care in Canada.^7,8^ As such, Indigenous people in Canada “all too often encounter health care systems that are not reflective of or grounded in cultural worldviews or definitions of health they uphold.”^2(pE208)^

In recent years, however, an increasing number of health care programs and services have emerged in Canada, which aim to enhance primary health care access and improve health outcomes for Indigenous Peoples.^2^ This trend aligns closely with the World Health Organization's Declaration of Astana,^9^ which emphasizes “knowledge and capacity building” in primary health care, including both biomedical and traditional—that is, Indigenous—knowledges. In the province of British Columbia (BC), this impulse is exemplified in the establishment of the First Nations Health Authority: a governmental partnership whereby First Nations communities increasingly regain control over their own health care services.^1(p32)^

Furthermore, an increasing number of Indigenous-led health services have arisen across Canada in recent years,^2^ “directed by the Indigenous communities that they are designed to serve.”^1(p31)^ Such programs are often explicitly decolonial—that is, antiracist and culturally safe—in character,^1,2^ and based on the principle of “culture as cure,” which centers “health interventions…[that] are holistic and informed by cultural knowledge or local spiritual worldviews,”^2(pE208)^ including Indigenous healing practices.

For non-Indigenous and biomedical health care providers working in such settings, “cultural humility”—a commitment to righting historical wrongs by “dismantling…power imbalances” and learning about Indigenous ways of knowing and doing^4(p9)^—is an essential ingredient.^2^

Aligned with the aforementioned decolonial principles, the present work offers a first scholarly account of the delivery of Chinese Medicine (CM) care on First Nations reserves in BC, Canada. With reference to the voices of clinicians working in the aforementioned settings, the study aims to: (1) develop a descriptive account of clinical operations; and, (2) explore operational successes and challenges across these clinics.

CM is a system of East Asian traditional (Indigenous) medicine with a pre-colonial, nonbiomedical epistemology informing the use of such traditional therapies as acupuncture and herbal medicine.^10^ Like other Indigenous therapeutic approaches, CM is internally diverse in terms of how its core epistemic principles are enacted in practice. Following the World Health Organization's guidance, many jurisdictions (including and beyond China) have standardized a particular, historically situated codification of Chinese medical knowledge known as “Traditional Chinese Medicine” into law. In Canada, BC is one such jurisdiction, where Traditional Chinese Medicine has been a statutorily regulated profession since 1996.^11^ While the present work refers to interviews with licensed practitioners of Traditional Chinese Medicine in BC, the authors will preferentially use the term “Chinese Medicine” (CM) throughout (except with reference to licensing and in direct quotes) to better recognize the diverse practice approaches employed by practitioners.

## Methods

Informed by a news article about the operation of CM clinics on First Nations reserves in BC, Canada,^12^ the authors received approval from the McMaster University Research Ethics Board to conduct qualitative interviews with CM and biomedical health care professionals working in such clinics. The first author contacted the lead clinician identified in the aforementioned news article, which led to the identification of a total of four clinics offering CM care in BC First Nations communities. Using a snowball sampling strategy, the authors aimed to secure English-language telephone interviews with lead clinicians working in each of these clinics. The authors constructed a semistructured interview guide with two broad areas of focus: identification of the clinics' operational characteristics; and, inquiry into clinician experiences working in these treatment settings.

Four health care practitioners, all fluent in English, consented to participate in the study: three licensed Traditional Chinese Medicine practitioners (two with East Asian ancestry; one of Middle Eastern ancestry) who were actively delivering care in BC First Nations communities; and, one biomedical health care professional (also a First Nations person who identified with the local community). Fieldwork and preliminary analyses for the present study constituted the first author's undergraduate thesis project at McMaster University, with the second author as her supervisor. This work represents a subsequent collaboration between the two authors.

The first author (a white-identified woman of mixed-European ancestry) conducted and transcribed telephone interviews with all respondents. Together with the second author (a woman of color with mixed South Asian and Western European ancestry, and a critical qualitative health researcher with clinical training in—and a scholarly focus on—East Asian medicine), the first author conducted a thematic analysis^13^ of all interview transcripts with reference to the study aims. Thematic analysis unfolded in two primary phases: a deductive coding process aimed at identifying key operational characteristics of each of the identified clinics; and, subsequently, an inductive coding process focused on characterizing clinician experiences and contextualizing these within the study's overarching decolonial framework. All coding and analytic decisions were reviewed and finalized by both coauthors.

In what follows, the authors present the study findings, using verbatim interview excerpts to illustrate and theoretically interpret key emergent themes. To protect respondent anonymity, the authors have elected to use the gender-inclusive pronoun “they” in all cases; but, when explicitly relevant, identify respondents according to their professional roles and/or ethnic ancestries. Overall, this article adheres to the guidelines articulated in the SRQR Reporting checklist for qualitative study.^14^

## Results

As shown in Table 1, the authors identified four distinct CM clinical programs (“a” through “d”) operating on First Nations reserves in BC, Canada. Two clinics (“a” and “b”) took place at existing community health centers where a single CM practitioner worked—in one case semiweekly and on a paid basis, in another, biweekly as a volunteer—alongside biomedical physicians, nurses, and pharmacists. At both centers, patients received CM care—acupuncture, herbal medicine, and lifestyle recommendations—free of charge. Operational costs were partially covered through funding partnerships with a nonprofit organization, supplemented in one case with monies from the First Nation Band Council (a local governing body), and in the other with a partial reimbursement from the provincial government's Medical Services Plan.

**Table 1.** Operational Overview of Chinese Medicine Clinics on First Nations Reserves in B.C., Canada

Clinical setting	Funding models	Treatments	Frequency
On reserve Community health center^a,b^ Band Council office^c^ School gymnasium^d^	Nonprofit agency donations^a,b,d^ Payment from Band Council^a,b,c^ Provincial Medical Service Plan^a,b,c^ Patient payment^c^	Acupuncture ^a,b,c,d^ Herbs^a,b,c^ Spiritually focused care^a,b,c,d^	Semiweekly^a,c^ Biweekly^b^ Annually^d^

Operational challenges			
Poorly suited clinical spaces	Chronic lack of funding	Herbal costs	Waitlists

The four studied clinics are differentiated in Table 1 using superscripts^a,b,c,d^.

A third semiweekly clinic (“c”), supported with funds from the Band Council and Medical Services Plan, operated as a “pop-up” clinic at a Band Council office wherein a single CM practitioner would set up (and take down) a treatment room on clinic days. There, patients received acupuncture treatments free of charge, but were asked to pay for any herbal medicines given. The fourth clinic (“d”), funded by the same nonprofit organization subsidizing operations at the two community health centers, operated on an annual basis at a school gymnasium, where 5 to 10 volunteer CM practitioners provided acupuncture, free of charge, to dozens of patients—separated with vertical dividers—over the course of a single day.

What follows is an exploration of three emergent study themes—Transculturalism, Cultural Humility, and Multidimensional Healing—followed by a discussion of challenges and future visions associated with the studied clinics.

### Transculturalism

Interviewed clinicians repeatedly indicated that it was not simply the technical use of acupuncture needles or herbal medicines that appeared to facilitate the acceptance of CM care within the First Nations communities served. Rather, they pointed to important transcultural similarities between the CM offered, and the beliefs held by community members. One respondent, a biomedical professional of First Nations ancestry, observes:

When [CM practitioners] first were introduced to our community, our community had to learn about Chinese medicine and their belief system. Once we thought there are huge similarities, especially about energy, we were like “yes.” …How they've explained their medicine-work is really similar to some of our Indigenous beliefs of how one heals. It's fairly universal in a lot of Indigenous communities to move energy in order to heal someone. For our Nation, the fact that they may use a needle whereas our Nation would use, could be a feather, or cedar, or cedar bows. We are healing people and not just physically but also spiritually.

One of the interviewed CM practitioners, a person of East Asian ancestry, provided tangible examples of such transculturalism in action:

One beautiful thing is the herb…So that's really important: the specific herbs [I use in clinic], and they [the patient] will say, “wow this works like, something that we use” … stimulati[ng] conversation that “my grandmother used to use this,” or “I am going to go and ask so-and-so what they use”—to be able to stimulate a conversation about the power and healing properties of our traditions… [People come in and we talk about] what can I do with my lifestyle to get healthier, how do I live off the land, how do I pay attention to where my food comes from? That's traditional for both of us.

Another CM practitioner—also a person with East Asian ethnic origins—spoke more conceptually about the potential for “culture-as-cure” to take place across systems of “old medicine, old knowledge”:

Chinese medicine…it's an old medicine… I think that there are ways of being as medicine people that are very transcultural. The old ways are the old ways, that's what is seems to me. Whether you are here, or you are there, they kind of transcend space, time, and dimension. They just are.

Transcultural exchange went both ways, with CM practitioners at times being “invited to the ceremonies” held on reserve, or receiving:

…small gifts, smoked salmon…sometimes hunters invite me over to pick up some meat on the way home, just gifts that people make to be able to say thank you, and say, you know, we hope this continues. That's nice, that's beautiful.

### Cultural humility

Interviewed CM practitioners consistently alluded to the concept of *cultural humility*, emphasizing their position as guests on First Nations reserves, serving at the community's lead rather than coming in to implement a therapeutic agenda of their own:

I think a lot about the concept of host and guest. I'm a guest on the land…This is an unceded territory. This is a Nation. That affects how I practice medicine. It is different to be the guest, and the Nation is the host. And within that there are a bunch of responsibilities… its everything from how one introduces oneself, it's calling your own ancestors into the land, it's acknowledging the ancestors of the land, the history of the land, it's acknowledging the relation of the history of the land with the history of that body.

Another practitioner shared:

As a practitioner I have to be always respectful and follow the lead of the First Nation. I can't go in and think that TCM [Traditional Chinese Medicine] is the next big thing. I can just walk in and say, this is who I am, what can I do to help? I can't assume, I have to be respectful of the leadership, following the direction of the First Nation, their level of readiness. If they invite me into the next step, and then I am part of a team that uses allopathic medicine, traditional Indigenous medicine. …It is not to take the lead or further colonize, that is not our role.

As another respondent indicated, the success of the CM care offered in a community health center setting furthermore depended on the cultural humility of the center's biomedical professionals, expressed in an open validation of a nonbiomedical therapeutic approach with community members:

We are fortunate that we have doctors that promote acupuncture with clients. I think it makes a difference for clients that really look to their family doctor for assurances that this is “ok” medicine.

Ultimately, clinicians' cultural humility appeared to support a therapeutic context in which healing could safely occur. One respondent shared that the Chief of the Nation where they worked indicated in a public speech that “the community feels safer since the TCM clinic began.” Importantly, it was also from patients that practitioners reported receiving positive feedback.

### Multidimensional healing

All interviewed CM practitioners characterized their work as providing multidimensional therapeutic benefits to patients served; healing, as they described it, went well beyond physical ailments to support improvements to mental and emotional health, as well as health at the community level. One clinician explained:

I have seen patients with arthritis who were in so much pain they didn't want to get out bed in the morning, [now] coming in with big smiles on their faces and hopping up on the table. A lot of elders with vertigo. Actually, two of them have told me they now go dancing. So, I see big changes to mental health, emotional health.

Practitioners repeatedly described clinical successes in using CM to treat health concerns where “community members…haven't really found a lot of success in Western medicine in treatment,” including reductions in patient usage of “narcotics [and] opioids.” As the interviewed biomedical clinician affirmed:

Chinese medicine looks at the whole person. …I do know that we have clients that have used Chinese medicine for their addiction issues. Also, I have seen success where clients decrease the amount of pain medication.

Respondents repeatedly reported that “once you have one person having success, then others are willing to try,” leading to greater acceptance of CM care within the broader community, with “people bringing their whole families in, from the elders to the children.” As the biomedical clinician indicated, such a context of trust building was an important component of health care work within the First Nations being served, given the legacies of mistrust for conventional biomedicine:

I have clients in the community who have not gone to Western medicine in 20 years because they have no faith in that system. And there is more than one, but I do know [one such] person goes to Chinese medicine and a Naturopath. And there are some here in the community that are actually becoming more open to that form of [non-biomedical] treatment. I mean you are looking at trauma, Indian residential schools, the way that Aboriginals were governed in this country, so there is a resistance in that older population.

CM practitioners similarly alluded to the complex, historically situated traumas that often underpinned patients' health challenges:

It's not just the superficial reasons. [Patients] come to see you for lower back pain, car accident, pain management. And once it is resolved, they keep coming and we see actually a lot of problems residing under this. It can be traced back to early childhood trauma, or tragedies in their family members' [lives].

At times, practitioners found it personally difficult to bear witness to the traumatic experiences reported by those they served:

Sometimes it is challenging with health and social situations, I have to remember to take care of myself. There are situations where I have to suck back tears because what people are telling me, the subjects get heavy.

Ultimately, however, all interviewed CM practitioners spoke to the gratifying character of their work, arising from the multidimensional healing that their work facilitated:

I had students doing herbal foot baths and massage of the feet, and I had an elder come in during one of my community events, and he started crying because his wife died twenty years ago, and he has actually not been touched kindly in twenty years. So, if that is not a reason to do the work, and to do really humble work, then I don't know what is.

### Operational challenges and future visions

Despite the many successes reported, all interviewed clinicians alluded to operational challenges (Table 1), the most notable of which was the economic insecurity represented by the clinics' reliance on external funding. The out-of-pocket costs to patients associated with providing herbal treatments furthermore limited the extent to which clinicians could recommend such medicines. Nevertheless, all respondents indicated, each in their own way, that the introduction of CM care within First Nations communities in BC had seemed successful, warranting consideration of further expansion. One CM practitioner indicated that some First Nations community members who had benefited from the studied programs “are getting [CM] training, and we are hoping that when they…obtain their license, they can go back to their community and give similar program[s] to them.” Importantly, the ethic of transculturalism and spirit of cultural humility discussed earlier permeated respondent reflections on how to move forward in future:

I am not going in saying that the answers are all in Traditional Chinese Medicine, that was never the point of this program. It was always: these are some traditions from somewhere else, from Indigenous people somewhere else, and why can't we grow together?

Ultimately, respondents tempered their vision of growing similar programs elsewhere with an emphasis on the agency of First Nations Peoples. As the interviewed biomedical clinician concludes, with reference to their own Nation:

Our song is our spirit, we believe that [it] gives us strength…And we believe that moving energy can cause [a] person to become better. That is a belief that has been here among Indigenous people here for 10,000 years. Chinese medicine, when they told me it unblocks energy so that a person's body sings, when it was described that way, I knew that I would be able to get my people to try it. And I think [that] is partly why it is successful here. I can't speak for other Indigenous populations. That is up to each community to share their song, their spirit, their way of being. …[But] I think if more communities—you know, ours was a pilot project—if more communities were given this opportunity, I think they would really benefit from that.

## Conclusion

The principles of transcultural exchange and cultural humility that emerged as central in the present study may provide useful parameters for similar projects in other Indigenous and ethnic minority communities detrimentally impacted by European colonization. Within such communities, where a historicized distrust for biomedicine is often endemic,^15^ the epistemic premises of Indigenous medical approaches from “outsider” Nations may—as suggested in this work—be experienced as culturally resonant. Importantly, the present study findings should not be construed as a broad endorsement of the potential value of acupuncture care in Indigenous or otherwise marginalized communities; instead, the reported success of the studied clinics may be more accurately attributed to the culturally humble and resonant manner in which CM-based acupuncture was delivered. Furthermore, the voices of First Nations community members who received on-reserve CM care as patients did not form part of the current study's scope, but should certainly be heard before making grand statements as to the studied programs' ostensible advantages.

Nevertheless, further investigation is warranted to explore the possible contributions of nonbiomedical, traditional medicine approaches as part of a broader strategy, aligned with the Declaration of Astana,^9^ to “improve health care outcomes and ensure access…[to] appropriate…care” for Indigenous and otherwise-marginalized communities in Canada and beyond.
